# Evaluating the Effectiveness of an Ultrasonic Acoustic Deterrent for Reducing Bat Fatalities at Wind Turbines

**DOI:** 10.1371/journal.pone.0065794

**Published:** 2013-06-19

**Authors:** Edward B. Arnett, Cris D. Hein, Michael R. Schirmacher, Manuela M. P. Huso, Joseph M. Szewczak

**Affiliations:** 1 Bat Conservation International, Austin, Texas, United States of America; 2 Forest and Range Experiment Station, United States Geological Survey, Corvallis, Oregon, United States of America; 3 Department of Biological Sciences, Humboldt State University, Arcata, California, United States of America; Università degli Studi di Napoli Federico II, Italy

## Abstract

Large numbers of bats are killed by wind turbines worldwide and minimizing fatalities is critically important to bat conservation and acceptance of wind energy development. We implemented a 2-year study testing the effectiveness of an ultrasonic acoustic deterrent for reducing bat fatalities at a wind energy facility in Pennsylvania. We randomly selected control and treatment turbines that were searched daily in summer and fall 2009 and 2010. Estimates of fatality, corrected for field biases, were compared between treatment and control turbines. In 2009, we estimated 21–51% fewer bats were killed per treatment turbine than per control turbine. In 2010, we determined an approximate 9% inherent difference between treatment and control turbines and when factored into our analysis, variation increased and between 2% more and 64% fewer bats were killed per treatment turbine relative to control turbines. We estimated twice as many hoary bats were killed per control turbine than treatment turbine, and nearly twice as many silver-haired bats in 2009. In 2010, although we estimated nearly twice as many hoary bats and nearly 4 times as many silver-haired bats killed per control turbine than at treatment turbines during the treatment period, these only represented an approximate 20% increase in fatality relative to the pre-treatment period for these species when accounting for inherent differences between turbine sets. Our findings suggest broadband ultrasound broadcasts may reduce bat fatalities by discouraging bats from approaching sound sources. However, effectiveness of ultrasonic deterrents is limited by distance and area ultrasound can be broadcast, in part due to rapid attenuation in humid conditions. We caution that an operational deterrent device is not yet available and further modifications and experimentation are needed. Future efforts must also evaluate cost-effectiveness of deterrents in relation to curtailment strategies to allow a cost-benefit analysis for mitigating bat fatalities.

## Introduction

As wind energy production has steadily increased worldwide, bat fatalities have been reported at wind facilities worldwide [1], [2], [3], [4] in a wide range of landscapes. A recent synthesis reported that approximately 650,000 to more than 1,300,000 bats have been estimated to have been killed from 2000–2011 in the U.S. and Canada [5]. Given these fatality rates, accelerating growth of the wind industry [6], and suspected and known population declines in many species of bats [7], [8], [9], it is imperative to develop and implement solutions to reduce future bat fatalities at wind facilities.

Prior studies have demonstrated that a substantial portion of bat fatalities consistently occur during relatively low-wind conditions over a relatively short period of time during the summer-fall bat migration period [2], [4]. Curtailment of turbine operations under these conditions and during this period has been proposed as a possible means of reducing impacts to bats [1], [2], [10]. Indeed, recent studies in Canada [11] and the U.S. [12] indicate that increasing turbine “cut-in speed” (i.e., wind speed at which wind-generated electricity enters the power grid) from the manufactured speed (usually 3.5–4.0 m/s for modern turbines) to between 5.0 and 6.5 m/s resulted in at least a 50% reduction in bat fatalities (and as high as 93%) compared to normally operating turbines [12]. While costs of lost power from curtailment can be factored into the economics and financing and power purchase agreements of new projects, altering turbine operations even on a partial, limited-term basis potentially poses operational and financial difficulties for existing projects, so there is considerable interest in developing other solutions to reduce bat fatalities that do not involve turbine shutdowns. Also, changing turbine cut-in speed may not be effective in other regions that experience bat fatalities although this strategy may ultimately prove sufficiently feasible and economical for reducing bat fatalities. Thus, research on alternative mitigation strategies and their associated costs are warranted.

Studies in Scotland suggest that bat activity may be deterred by electromagnetic signals from small, portable radar units [13]. This study reported that bat activity and foraging effort per unit time were significantly reduced during experimental trials when their radar antenna was fixed to produce a unidirectional signal that maximized exposure of foraging bats to their radar beam. The effectiveness of radar as a potential deterrent has not been tested at an operating wind facility to determine if bat fatalities could be significantly reduced by these means. Moreover, the effective range of electromagnetic signals as well as the number of radar units needed to affect the most airspace near individual turbines would need to be determined to fully evaluate effectiveness and to allow some cost-benefit analysis relative to other potential deterrents or curtailment [11], [12].

Echolocating bats produce high frequency vocal signals and perceive their surroundings by listening to features of echoes reflecting from targets in the path of the sound beam [14]. Thus, bats that use echolocation depend heavily on auditory function for orientation, prey capture, communication, and obstacle avoidance. Bats of some species avoid certain territorial social calls emitted by conspecifics [15] and are deterred by “clicks” emitted by noxious moths [16]. Because echolocating bats depend upon sensitive ultrasonic hearing, we hypothesized that broadcasting ultrasound from wind turbines may disrupt or “jam” their perception of echoes and serve as a deterrent. Such masking of echo perception, or simply broadcasting high intensity sounds at a frequency range to which bats are most sensitive, could create an uncomfortable or disorienting airspace that bats may prefer to avoid.

Few studies have investigated the influence of ultrasound broadcast on bat behavior and activity, particularly under field conditions. Broadband random ultrasonic noise may mask bat echolocation somewhat, but not completely [17]. Ultrasound broadcasts can reduce bat activity, perhaps due to greater difficulty in the bats hearing echoes of insects and thus reduced feeding efficiency [18]. A laboratory test of the response of big brown bats (*Eptesicus fuscus*) to a prototype eight speaker deterrent device emitting broadband white noise at frequencies ranging from 12.5–112.5 kHz in the laboratory and found that during non-feeding trials, bats landed in a quadrant containing the device significantly less when it was broadcasting broadband noise (J. Spanjer, University of Maryland and E. Arnett, Bat Conservation International, unpublished data). During feeding trials in this experiment, bats never successfully captured a tethered mealworm when the device broadcasted sound but captured mealworms near the device in about 1/3 of trials when it was silent. Field tests of the same acoustic deterrent found that when placed by the edge of a small pond, where nightly bat activity was consistent, nightly activity decreased significantly on nights when the deterrent was activated (J. Szewczak, Humboldt State University and E. Arnett, Bat Conservation International, unpublished data).

Our goal was to improve deterrent devices we previously developed and tested by increasing the effective area of ultrasonic emissions from the nacelle of wind turbines, and to test their effectiveness on reducing bat fatalities. The objectives of this study were 1) to conduct carcass searches and field bias trials (searcher efficiency and carcass removal) to determine rate of bat fatalities at treatment (those with deterrent devices) and control turbines; and 2) compare bat fatality rates at turbines treatment and control turbines to determine effectiveness. We successfully tested our ultrasonic deterrent device at an operating wind facility and offer suggestions for future efforts regarding this potential mitigation strategy to reduce bat fatalities at wind facilities.

## Methods

### Study Area

The study was conducted at the Locust Ridge Wind Project located near the towns of Shenandoah, Mahanoy City, and Brandonville in Columbia and Schuylkill Counties, Pennsylvania, and consisted of two different facilities. The Locust Ridge I (LRI) Wind Farm has 13 Gamesa G87 2.0 MW turbines, each on 80 m monopoles with a rotor diameter of 87 m and a swept area of 5,945 m^2^. There were 51 Gamesa G83 2.0 MW turbines, each on 80 m monopoles with a rotor diameter of 83 m and a swept area of rotor-swept area of 5,411 m^2^, at the Locust Ridge II (LRII) Wind Farm. The facilities lie within the Appalachian mixed mesophytic forests ecoregion and the moist broadleaf forests that cover the plateaus and rolling hills west of the Appalachian Mountains [19], [20]. Elevations along ridges where turbines are located range from 530–596 m. All turbines were located along a ridge in deciduous forest, with some species of evergreen trees interspersed. Vegetation across the area included thickets of scrub oak (*Quercus berberidifolia*) interspersed with chestnut oak (*Quercus prinus*) and gray birch (*Betula populifolia*), and mature hardwood forests of red oak (*Quercus rubra*), red maple (*Acer rubrum*), yellow birch (*Betula alleghaniensis*), American beech (*Fagus grandifolia*) and scrub oak.

### Turbine Selection and Deterrent Installation

We randomly selected 15 of the 51 turbines located at LR II to be searched as part of a separate study to determine post-construction fatality rates and to meet permitting requirements of the Pennsylvania Game Commission’s voluntary agreement for wind energy [21]. These 15 turbines were our control turbines for comparing with treatment turbines, those fitted with deterrent devices. In 2009, unforeseen mechanical and safety issues arose at the LRII site and many of these turbines had to be excluded from our potential treatment group due to potential safety hazards. Thus, we included all 13 turbines at LRI as well as the remaining available turbines at LRII (n = 36) when randomly selecting our 10 turbines to be fitted with deterrent devices; 3 turbines were randomly selected from the 13 available at the LRI site and 7 of 36 available at LRII.

We did not assess whether there were any potential inherent differences between the two types of turbines in 2009 and for this year assumed there were no confounding differences in our findings. However, in 2010, we attempted to assess inherent differences between control and treatment turbines by modifying our design and analysis to reflect a Before-After Control-Impact (BACI) design. The same sets of control and treatment turbines were monitored for a period of time prior to implementation of the deterrent treatment (1 May to 26 July 2010), then again during the deterrent implementation period (31 July through 9 October 2010). This design allowed for incorporating initial inherent differences between the two experimental treatment sets prior to implementation of the treatment as a reference for interpreting any differences detected during implementation of the treatment.

The deterrent devices used in our study consisted of a waterproof box (∼45×45 cm, ∼0.9 kg) that housed 16 transducers that emitted continuous broadband ultrasound from 20–100 kHz (manufactured by Deaton Engineering, Georgetown, Texas; Figure S1). We did not test other types of emissions (e.g., short pulses) concurrent with broadband emission because more devices and sample turbines would be required, thus resulting in cost and sample size constraints. Transducers we used had an optimum transmission level at their resonant frequency of 50 kHz transmission and reduced transmit levels at higher and lower frequencies over a broadband range of 20–100 kHz. This frequency range overlaps that of all bats known in the study area. Three factors influence the predicted effective transmitted power at a given distance: 1) the original transmitted power (sound pressure level; SPL); 2) attenuation with distance due to the wave front spreading (inversely proportional to the square of the distance, frequency independent); and 3) attenuation (absorption) in air of the sound wave (dependent on frequency, humidity and distance; Tables S1 and S2). The following discussion describes our estimation to base the target signal level of our deterrent:

A typical bat emits calls at about 110 dB sound pressure level (SPL) at 10 cm [22]. During search phase flight a typical North American species of bat emits about 12 calls per second, each about 5 milliseconds in duration [23], [24]. Given the speed of sound at 340 m/sec and duration of an open air call, the bat’s own call will theoretically mask echoes returning from objects within about 1.5 m (i.e., the bat cannot hear early return echoes while vocalizing). An echo from a target about 1.5 m away will return about 45 dB less than the original 110 dB signal, or at about 65 dB. The bat’s next call would mask echoes returning from about 25 m away. By this first order estimation, a bat would theoretically perceive information from returning echoes with amplitudes of ≤65 dB over a range from about 1.5–25 m. Thus, we estimated that a broadband signal of ≥65 dB would begin jamming or masking most bat’s echo perception from targets beyond about a 1.5 m range.

We attached 8 individual deterrent devices to the nacelle of each of 10 sample turbines. Three devices on each side of the nacelle were evenly spaced and pointed downward with one aimed into the rotor-swept area, one parallel with the monopole, and one aimed toward the back of the nacelle (Figures S2 and S3). Additionally, two devices were aimed at reflector plates; one that projected emissions into the upper part of the rotor-swept area, and one toward the rear of the nacelle. All devices connected to control boxes that were powered from outlets located in the nacelle and each was set on a timer to operate from ½ hour before sunset to ½ hour after sunrise each night of the study.

### Delineation of Carcass Search Plots and Habitat Mapping

We delineated a rectangular plot 126 m north-south by 120 m east-west (60 m radius from the turbine mast in any direction; 15,120 m^2^ total area) centered on each turbine sampled; this area represents the maximum possible search area for this study. Transects were set 6 m apart within each plot and in an east-west direction, due to the topography and layout of turbines at this facility. However, dense vegetation and the area cleared of forest at this facility was highly varied and, thus, we eliminated unsearchable habitat (e.g., forest) and usually did not search the entire possible maximum area. We used a Trimble global positioning system (GPS) to map the actual area searched at each turbine. The density-weighted area searched was used to standardize results and adjust fatality estimates (see statistical methods). The habitat visibility classes within each plot were also mapped using a GPS unit. We recorded the percent ground cover, height of ground cover (low [<11 cm], medium [11–50 cm], high [>50 cm]), type of habitat (vegetation, brush pile, boulder, etc), and the presence of extreme slope and collapsed these habitat characteristics into visibility classes that reflect their combined influence on carcass detectability (Table S3) [21].

### Fatality Searches

We conducted daily searches at 15 control turbines and 10 treatment turbines from 15 August to 10 October 2009 and 1 May to 26 July and 31 July to 9 October 2010. Each searcher completed 5–7 turbine plots each day during the study. Searchers walked at a rate of approximately 10–20 m/min along each transect searching out to 3 m on each side for fatalities. Searches were abandoned only if severe or otherwise unsafe weather (e.g., heavy rain, lightning) conditions were present and searches were resumed that day if weather conditions permitted. Searches commenced at sunrise and all turbines were searched within 8 hr after sunrise.

We recorded date, start time, end time, observer, and weather data for each search at turbines. When a dead bat or bird was found, the searcher placed a flag near the carcass and continued the search. After searching the entire plot, the searcher returned to each carcass and recorded information on date, time found, species, sex and age (where possible), observer name, identification number of carcass, turbine number, perpendicular distance from the transect line to the carcass, distance from turbine, azimuth from turbine, habitat surrounding carcass, condition of carcass (entire, partial, scavenged), and estimated time of death (e.g., ≤1 day, 2 days, etc.). A field crew leader confirmed all species identifications at the end of each day. Disposable nitrile gloves were used to handle all carcasses to reduce possible human scent bias for carcasses later used in scavenger removal trials. Each carcass was placed into a separate plastic bag and labeled. Fresh carcasses, those determined to have been killed the night immediately before a search, were redistributed at random points on the same day for searcher efficiency and scavenging trials.

### Ethics Statement

This study was conducted on private property and authorized by the operator Iberdrola Renewables Locust Ridge Wind LLC. All downed bats were euthanized, even if no physical injury was observed due to the possibility of barotraumas, following requirements by the Pennsylvania Game Commission protocol [21] and using acceptable methods suggested by the American Society for Mammalogists [25]; because sedation or anesthesia was not used in our study, we employed cervical dislocation. Our work did not require approval by an Institutional Animal Care and Use Committee and all aspects of the field work and permission to collect carcasses found during our study was conducted under the auspices of permits issued each year by the state of Pennsylvania, Pennsylvania Game Commission, and the U.S. Fish and Wildlife Service.

### Field Bias Trials

Searcher efficiency and removal of carcasses by scavengers (herein referred to as carcass persistence) was quantified to adjust estimates of total bat and bird fatalities for detection bias. We conducted bias trials throughout the entire study period and searchers were never aware which turbines were used or the number of carcasses placed beneath those turbines during trials. Prior to the study’s inception, we generated a list of random turbine numbers and random azimuths and distances (m) from turbines for placement of each bat used in bias trials.

We used only fresh killed bats for searcher efficiency and carcass removal trials during the study. At the end of each day’s search, a field crew leader gathered all carcasses from searchers and then redistributed fresh bats at predetermined random points within any given turbine plot’s searchable area. Data recorded for each trial carcass prior to placement included date of placement, species, turbine number, distance and direction from turbine, and visibility class surrounding the carcass. We attempted to distribute trial bats equally among different visibility classes throughout the study period and succeeded in distributing roughly one-third of all trial bats in each visibility class (easy, moderate, and difficult; difficult and very difficult were combined). We attempted to avoid “over-seeding” any one turbine with carcasses by placing no more than 4 carcasses at any one time at a given turbine. Because we used fresh bats for searcher efficiency trials and carcass removal trials simultaneously, we did not mark bats with tape or some other previously used methods [26] that could impart human or other scents on trial bat carcasses. Rather, we used trial bat placement details (i.e. azimuth, distance, sex, species) and signatures from hair and tissue samples (i.e., hair removed between the scapulae and wing punches) to distinguish them from other fatalities landing nearby. Each trial bat was left in place and checked daily by the field crew leader or a searcher not involved with the bias trials at turbines where carcasses were placed. Thus, trial bats were available to be found by searchers on consecutive days during daily searches unless removed by a scavenger. We recorded the day that each bat was found by a searcher, at which time the carcass remained in the scavenger removal trial. If, however, a scavenger removed a carcass before detection it was removed from the searcher efficiency trial and used only in the removal data set. When a bat carcass was found, the searcher determined if a bias trial carcass had been found by looking for markings described above and contacting the crew leader to determine if the location (direction and distance) matched any possible trial bats. All trial bats were left in place for the carcass removal trial. Carcasses were left in place until removed by a scavenger or they decayed and disintegrated to a point beyond recognition. Carcass condition was recorded daily up to 20 days, as present and observable or missing or no longer observable.

### Statistical Methods

#### Carcass persistence/removal

Estimates of the probability that a bat carcass was not removed in the interval between searches were used to adjust carcass counts for removal bias. Removal included scavenging, wind or water, or decomposition beyond recognition. In most fatality monitoring efforts, it is assumed that carcass removal occurs at a constant rate that is not dependent on the time since death; this simplifying assumption allows us to estimate fatality when search intervals exceed one day. The length of time a carcass remains on the study area before it is removed is typically modeled as an exponentially distributed random variable. The probability that a carcass is not removed during an interval of length *I* can be approximated as the average probability of persisting given its death might have occurred at any time during the interval:
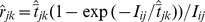
where:


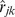
 is the estimated probability that a carcass in the *k* ^th^ visibility class that died during the interval preceding the *j* ^th^ search will not be removed by scavengers;


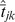
 is the estimated average persistence time of a carcass in the *k* ^th^ visibility class that died during the interval preceding the *j* ^th^ search;


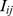
 is the length of the effective interval preceding the *j* ^th^ search at the *i* ^th^ turbine;

Data from 351 and 408 bat carcasses in 2009 and 2010, respectively, were used in our analysis, with carcass persistence time modeled as a function of visibility class. We fit carcass persistence/removal data for bats to an interval-censored parametric failure time model, with carcass persistence time modeled as a function of size and/or visibility class. We used a relatively liberal alpha of 0.15 to identify factors (e.g., carcass size, visibility classes) that influence bias parameter values (i.e., searcher efficiency and carcass persistence) for removal of bat carcasses.

#### Searcher efficiency

Estimates of the probability that an observer will visually detect a carcass during a search were used to adjust carcass counts for observer bias. Failure of an observer to detect a carcass on the search plot may be due to its size, color, or time since death, as well as conditions in its immediate vicinity (e.g., vegetation density, shade). In most fatality monitoring efforts, because we cannot measure time since death, it is assumed that a carcass’ observability is constant over the period of study, which it likely is not. In this study, searches were conducted daily and carcass persistence times were long, providing an opportunity for a searcher to detect a carcass that was missed on a previous search. We used a newly derived estimator [27] that assumes a carcass missed on a previous search will not be observed on a subsequent search (i.e., there are inherent environmental conditions that make the carcass unobservable like heavy foliage, terrain). If this assumption is not met, it can lead to overestimates of fatality. Other estimators [26] assume that a carcass missed on a previous search has the same probability of being observed as it had on the first search (i.e., there is nothing inherent in the environment surrounding the carcass that makes it unobservable), missing it is purely a chance event and that if the carcass is not removed by predators and enough searches are conducted, it will eventually be observed. If this assumption is not met, it can lead to underestimates of fatality. It is likely that neither assumption is appropriate in all cases.

Searcher efficiency trial carcasses were placed on search plots and monitored for 20 days. The day on which a bat carcass was either observed or removed by a scavenger was noted. In these trial data, if a carcass had not been found within the first 8 searches it had essentially no chance of being found. This lends empirical support to the idea that there are some environmental conditions surrounding the carcass that determine its probability of being found. However, several carcasses missed on the first search were found on subsequent searches, lending support to the idea that at least for some carcasses, the probability of missing them is purely a chance event. To allow for some possibility of observing a carcass once having missed it, the set of trial carcasses comprised those found or still observable but not found within the first 8 searches. After accounting for carcasses removed before a searcher had the chance of observing them, we fit data from 139 (2009) and 169 (2010) bat carcasses to a logistic regression model, with odds of observing a carcass given that it persisted, modeled as a function of visibility class. Again, we used a relatively liberal alpha of 0.15 to determine if a significant effect among visibility classes existed. Because we found no bats in the very difficult visibility class, SE was not modeled for this class.

#### Density of carcasses and proportion of area surveyed

Density of carcasses is known to diminish with increasing distance from the turbine [26], so a simple adjustment to fatality based on area surveyed would likely lead to overestimates, because unsearched areas tend to be farthest from turbines where carcass density is lowest. The calculated function (see below) relating density to distance from a turbine was used to weight each square meter in the plot. The density-weighted fraction of each plot that was actually searched was used as an area adjustment to per-turbine fatality estimates rather than using a simple proportion.

The density of bat carcasses (number of carcasses/m^2^) was modeled as a function of distance (m) from the turbine. Because searcher efficiency and visibility class are confounded with distance, only fresh bat carcasses found in Easy visibility class were used for this analysis and all non-incidental data from all searched turbines were used, yielding a total of 172 fresh bat carcasses. We assumed that the carcass persistence time and searcher efficiency would be equal for all carcasses within this class and would not change as a function of distance from the turbine. We also assumed that no bat carcasses killed by turbine blades would fall >200 m from the turbine. Carcasses were “binned” into 2 m rings extending from the turbine edge out to the theoretical maximum plot distance (Figure S4). We determined the total area among all search plots that was in the easy visibility class (m^2^) in each ring and calculated carcass density (number of carcasses/m^2^) in each ring. Density was modeled as a conditional cubic polynomial function of distance (dist):

If distance ≤50 m, then density = exp (−1.77328+0.0346454*dist −0.00271076* dist^2^+0.0000229885* dist^3^ ) − 0.01, else density = 0.009363847*exp (−0.05*(distance-50)).

Relative density was derived by dividing the predicted density of each m^2^ unit by the total predicted density within 200 m of a turbine, providing a density-weight for each m^2^ unit. The density weighted area (DWA) of a plot was calculated as the sum of the density weights for all m^2^ units within the searchable area. If no portion of a designated plot was unsearchable, the density weight for the plot would be 1. The physical area surveyed within a plot differed among turbines and ranged from 20–47% of the delineated theoretical maximum search plot, with an average of 31% whereas the weighted density area of plots averaged 62% (range: 44–78%). In addition, using this density weight, we estimated 7.2% of the carcasses killed at a turbine would be found beyond the boundaries of the designated search plot.

#### Fatality estimates

We adjusted the number of bat fatalities found by searchers by estimates of searcher efficiency and by the proportion of carcasses expected to persist unscavenged during each interval using the following equation:
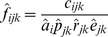
where:


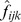
 is the estimated fatality in the *k* ^th^ visibility class that occurred at the *i* ^th^ turbine during the *j* ^th^ search;


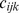
is the observed number of carcasses in the *k* ^th^ visibility class at the *i* ^th^ turbine during the *j* ^th^ search;


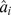
is the density-weighted proportion of the area of the *i* ^th^ turbine that was searched;


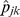
is the estimated probability that a carcass in the *k* ^th^ visibility class that is on the ground during the *j* ^th^ search will actually be seen by the observer;


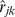
is the probability than an individual bird or bat that died in the *k* ^th^ visibility class during the interval preceding the *j* ^th^ search will not be removed by scavengers; and


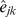
is the effective interval adjustment (i.e., the ratio of the length of time before 99% of carcasses can be expected to be removed to the search interval) associated with a carcass in the *k* ^th^ visibility class that died during the interval preceding the *j* ^th^ search.

The value for 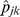
was estimated through searcher efficiency trials with estimates given above; 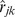
is a function of the average carcass persistence rate and the length of the interval preceding the *j* ^th^ search; and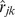, 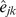 and 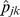 are assumed not to differ among turbines, but differ with search interval (*j)* and visibility class (*k*).

The estimated annual per turbine fatality for bats and birds was calculated using a newly derived estimator [27] and the equation is:
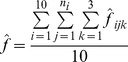
where *n*_i_ is the number of searches carried out at turbine *i*, 1 = 1, …, 10, and 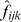
is defined above. The per turbine estimate and confidence limits were multiplied by 64, the total number of turbines, and divided by 0.9279 to adjust for actual density-weighted area searched to give total annual fatality estimates [28]. This estimate assumes that no fatalities occurred during the winter, i.e. prior to April and after November. No closed form solution is yet available for the variance of this estimator, so 95% confidence intervals of this estimate were calculated by bootstrapping [29]. Searcher efficiency was estimated from a bootstrap sample (with replacement) of searcher efficiency data, carcass persistence estimated from a bootstrap sample of carcass persistence data, and these values were applied to the carcass data from a bootstrap sample of turbines to estimate average fatality per turbine. This process was repeated 1000 times. The 2.5^th^ and 97.5^th^ quantiles from the 1,000 bootstrapped estimates formed the 95% confidence limits of the estimated fatality [27].

#### Comparison between treatment and control turbines

In 2009, we compared average fatality at control with treatment turbines for all bats and for each species using one-way analysis of variance with each turbine as the experimental unit and log_e_ transformed estimated total fatalities as the response. In 2010, estimated average bat fatality per turbine at control and treatment turbines, during the treatment phase and the period immediately preceding it (pre-treatment phase) was analyzed using a BACI approach [26], [30], [31], employing ANOVA repeated measures with the turbine as the experimental unit, repeatedly measured twice. Our approach determined whether the ratio of average per-turbine fatality at control turbines (n = 15) to treatment turbines (n = 10) during implementation of the deterrents was significantly greater than it was in the period immediately preceding implementation of the treatments. In both years, the fatality data were log transformed to satisfy assumptions of normality and homogeneity of variance [32].

## Results

In 2009, we searched 15 control turbines and 10 treatment turbines each day between 15 August and 10 October, and did not assess inherent variability among turbines. We found 194 carcasses (135 at control, 59 at treatment) of 6 species and two carcasses were not identifiable to species in 2009 (Table 1). During the pre-treatment period between 1 May and 26 July 2010, we searched 15 control turbines daily for all but 2 days (16 May and 2 June) and 10 Deterrent turbines daily for all but 4 days (9, 20, 24 25 July 2010) due to heavy rain, or facility maintenance. During the treatment period between 1 August and 15 October, we searched 15 control turbines daily for all but 4 days (26 August; 22, 29, 30 September 2010) and 10 Deterrent turbines daily for all but 3 days (19 August; 9, 30 September 2010) due to heavy rain or facility maintenance. During the pre-treatment period from 1 May to 26 July 2010, we found 59 carcasses comprising 6 species of bats (37 at control, 22 at treatment; Table 2). During the treatment period, we found 223 carcasses comprising 6 species of bats (162 at control, 61 at Deterrent; Table 3). Fatalities were found at all 25 turbines searched and time required to search each plot ranged from 12–100 minutes in both years of the study. Based on data from turbines not equipped with deterrents, the estimated fatality rate for this site ranged from 16–29.3 bats/turbine/year (8–14.7/MW/year) from 2009–2010.

**Table 1:** Number of bats by species and age/sex class found under turbines at the Locust Ridge Wind Project, Columbia and Schuylkill Counties, Pennsylvania, 1 April–15 November 2009.

2009						
	Adult	Adult	Juvenile	Juvenile		
	male	female	male	female	Unknown	Total
**Control**						
Big brown	3	–	2	3	2	**10**
Eastern red	6	2	1	–	4	**13**
Hoary	11	8	2	3	6	**30**
Little brown	12	2	6	2	2	**24**
Silver-haired	12	8	3	2	1	**26**
Tri-colored	12	2	8	5	4	**31**
Unknown	–	–	–	–	1	**1**
Sub-total	*56*	*22*	*22*	*15*	*20*	***135***
**Treatment**						
Big brown	1	–	2	–	1	**4**
Eastern red	2	3	1	2	1	**9**
Hoary	6	1	–	1	2	**10**
Little brown	9	2	1	–	1	**13**
Silver-haired	1	1	–	1	5	**8**
Tri-colored	3	2	2	4	2	**13**
Unknown	–	–	–	–	2	**2**
Sub-total	*22*	*9*	*6*	*8*	*14*	***59***
**Total**	**78**	**31**	**28**	**23**	**34**	**194**

**Table 2:** Number of bats by species and age/sex class found under turbines at the Locust Ridge Wind Project, Columbia and Schuylkill Counties, Pennsylvania, 1 May–26 July (Pre-experiment phase).

2010 Pre-treatment period (1 May–26 July)						
	Adult	Adult	Juvenile	Juvenile		
	male	female	male	female	Unknown	Total
**Control**						
Big brown	5	1	–	–	2	**8**
Eastern red	4	7	–	–	–	**11**
Hoary	6	4	–	–	1	**11**
Little brown	1	2	–	–	–	**3**
Silver-haired	1	1	–	–	–	**2**
Tri-colored	2	–	–	–	–	**2**
Unknown	–	–	–	–	–	**–**
Sub-total	*19*	*15*	*–*	*–*	*3*	***37***
**Treatment**						
Big brown	5	1	–	–	–	**6**
Eastern red	6	1	–	–	–	**7**
Hoary	4	1	–	1	1	**7**
Little brown	–	–	–	–	–	**–**
Silver-haired	–	–	–	–	–	**–**
Tri-colored	2	–	–	–	–	**2**
Unknown	–	–	–	–	–	**–**
Sub-total	*17*	*3*	*–*	*1*	*1*	***22***
**Total**	**36**	**18**	**0**	**1**	**4**	**59**

**Table 3:** Number of bats by species and age/sex class found under turbines at the Locust Ridge Wind Project, Columbia and Schuylkill Counties, Pennsylvania, 31 July–9 October (experiment phase) 2010.

2010 Treatment period (31 July–9 August)						
	Adult	Adult	Juvenile	Juvenile		
	male	female	male	female	Unknown	Total
**Control**						
Big brown	2	4	2	1	–	**9**
Eastern red	28	19	–	–	3	**50**
Hoary	32	10	4	4	11	**61**
Little brown	6	–	–	–	–	**6**
Silver-haired	9	10	–	–	1	**20**
Tri-colored	8	2	1	1	4	**16**
Unknown	–	–	–	–	–	**–**
Sub-total	*85*	*45*	*7*	*6*	*19*	***162***
**Treatment**						
Big brown	1	–	–	–	–	**1**
Eastern red	9	10	–	–	3	**22**
Hoary	11	6	–	2	3	**22**
Little brown	1	1	–	–	1	**3**
Silver-haired	1	1	1	–	2	**5**
Tri-colored	2	2	1	–	3	**8**
Unknown	–	–	–	–	–	**–**
Sub-total	*25*	*20*	*2*	*2*	*12*	***61***
**Total**	**110**	**65**	**9**	**8**	**31**	**223**

### Fatality Estimates in 2009

A total of 278 trial carcasses were used to estimate searcher efficiency in this study. One hundred thirty-nine of the 145 (96%) carcasses in the easy class that persisted >7 days were found by searchers, while 105 of the 123 (85%) carcasses in the moderate class that persisted long enough to be observed were found. Eight of 10 (80%) carcasses in the difficult class were found. A logistic regression model of the odds of detection given persistence as a function of visibility classes was fit to the data and there was strong evidence of a difference in searcher efficiency among the visibility classes (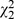 = 10.32, *p = *0.006). Data from 351 scavenger removal trial carcasses were fit to an interval-censored parametric failure time model. Average carcass persistence time was found to be strongly related to visibility classes (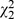 = 6.58, *p = *0.037). Average persistence time was estimated to be 9.4 days (95% CI: 7.7, 11.7 days), 13.9 days (95% CI: 10.8, 18.3 days) and 8.7 days (95% CI: treatment 4.6, 16.1 days) in easy, moderate and difficult visibility classes respectively. Estimates of the probability of a bat carcass persisting for 1 day (*r*) were 0.948 (95% CI: 0.938, 0.958), 0.964 (95% CI: 0.955, 0.973) and 0.942 (95% CI: 0.900, 0.970), respectively.

The average per-turbine fatality rate at treatment turbines was significantly less than at control turbines (*F*_1,23_ = 14.7, *p<*0.001). We estimated an average of 11.6 bats (95% CI: 9.4, 14.1) were killed per turbine at treatment turbines during this period, compared to 18.4 bats (95% CI: 16.0, 21.3) killed per turbine at control turbines. We estimated 60% higher fatality (95% CI: 26%, 104%) per control turbine than per treatment turbine from 15 August to 10 October 2009, or conversely, 21–51% estimated fewer bats were killed per treatment turbine than per control turbine.

We estimated twice as many hoary bats (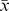 = 2.09, 95% CI = 1.18, 4.04) killed per control turbine than treatment turbine, and nearly twice as many silver-haired bats (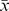
1.88, 95% CI = 0.92, 5.14), although the estimated effect was not significant for this species (Tables 3 and 4). Results for other species were highly variable with no statistically significant difference between turbine groups (Tables 4 and 5).

**Table 4:** Number of each species found (N) and the estimated bat fatalities/turbine (mean and 95% confidence intervals [CI]) for each species of bat per turbine, adjusted for searcher efficiency, carcass removal, and area, at control and treatment turbines at the Locust Ridge Wind Project in Columbia and Schuylkill Counties, Pennsylvania, 15 August–10 October 2009.

		Control Turbines				Treatment Turbines		
*Species*	N	Mean	Lower 95% CI	Upper 95% CI	N	Mean	Lower 95% CI	Upper 95% CI
Big brown bat	10	1.34	0.35	2.59	4	0.78	0.20	1.36
Eastern red bat	13	1.81	0.95	2.83	9	1.73	0.73	2.73
Hoary bat	30	4.14	3.13	5.19	10	1.98	1.12	3.22
Little brown bat	24	3.36	2.14	5.05	13	2.66	1.57	3.82
Silver-haired bat	26	3.51	2.08	4.98	9	1.85	0.75	3.27
Tri-colored bat	31	4.15	2.36	6.20	13	2.47	1.29	3.99
Unknown bat	1	0.12	0.10	0.48	1	0.17	0.16	0.51

**Table 5:** Ratio between bat fatalities per control turbine relative to treatment turbines (mean and 95% confidence intervals [CI]) for each species of bat from the Locust Ridge Wind Project in Columbia and Schuylkill Counties, Pennsylvania, 15 August–10 October 2009.

Species	Mean Ratio control:treatment	Lower 95% CI	Upper 95% CI
Big brown bat	1.74	0.41	6.13
Eastern red bat	1.06	0.44	2.75
Hoary bat*	2.09	1.18	4.04
Little brown bat	1.27	0.71	2.36
Silver-haired bat	1.88	0.92	5.14
Tri-colored bat	1.68	0.80	3.58
Unknown bat	0.12	0.00	2.28

Confidence intervals that do not include 1.0 are considered statistically significant (*).

### Fatality Estimates in 2010

A total of 169 bat carcasses were used to estimate searcher efficiency in this study. Eighty three of 86 (97%) carcasses in the easy class that persisted >7 days were found by searchers, while 59 of 70 (84%) carcasses in the moderate class that persisted long enough to be observed were found. Eight of 13 (62%) carcasses in the difficult class were found. Because no fatalities were found in the very difficult class, we removed the 6 bats placed in this class from our analysis. A logistic regression model of the odds of detection given persistence was fit to the visibility classes and there was strong evidence of a difference in searcher efficiency among the visibility classes (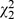 = 14.59, *p = *0.007).

Data from 408 scavenger removal trial carcasses were fit to an interval-censored parametric failure time model. Average carcass persistence time was found not to be related to visibility class (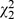 = 0.56, *p = *0.907), but there was moderate evidence that average persistence time was longer before the treatment period than during the treatment period (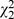 = 4.27, *p = *0.12). Average persistence time was estimated to be 7.8 days (95% CI: 6.4, 9.6 days) prior to implementation of the treatments and 6.2 days (95% CI: 5.4, 7.1 days) during the implementation of the treatments. This slight difference in average persistence time had little effect on the probability of a carcass persisting through the search interval. The estimated probability of a bat carcass persisting for 1 day (*r*) was 0.939 (95% CI: 0.926, 0.950) prior to the treatment period and 0.923 (95% CI: 0.912, 0.933) during the treatment period.

Bat fatality data from the pre-treatment period were used to evaluate if there were inherent difference between control and treatment turbines. We determined there was marginal evidence that the ratio of control:treatment fatalities was greater during the treatment period than in the pre-treatment period (*F*_1,23_ = 3.9, p = 0.061). During the pre-treatment period, prior to implementation of the deterrents, fatality per control turbine was estimated to be 1.09 times greater than per treatment turbine (95% CI: 0.74–1.61). We determined the initial inherent difference was about 9% in the fatality rate between the two sets and, while this was not statistically significant, we chose to adjust our comparison of fatalities between control and treatment turbines accordingly.

During the treatment period, we estimated an average of 12.8 bats (95% CI: 9.5, 17.2) were killed per turbine at treatment turbines compared to 22.9 bats (95% CI: 18.0, 29.3) killed per turbine at control turbines. Bat fatalities per control turbine was estimated to be 1.8 times greater than per treatment turbine (95% CI: 1.22–2.64); in other words, 18–62% fewer bats killed per treatment turbines relative to control turbines during the treatment period. As stated above, however, we determined an approximate 9% inherent difference between treatment and control turbines and fatality per control turbine was estimated to be 1.09 times greater than per treatment turbine (95% CI: 0.74–1.61) prior to implementation of the treatment. Thus, the ratio of fatality per control turbine relative to treatment turbines after implementing the treatment was estimated to be 1.64 times greater than the pre-treatment period ratio (95% CI: 0.98, 2.76). In other words, between 2% more and 64% fewer bats were killed per treatment turbine relative to control turbines after accounting for inherent turbine differences prior to treatment implementation.

In 2010, prior to implementation of the deterrent treatment, we estimated 1.47 times as many hoary bats (95% CI = 0.39, 3.42) and 1.32 times as many silver-haired bats (95% CI = 0.47, 3.27) killed per control turbine than treatment turbine. Although we estimated nearly twice as many hoary bats (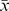 = 1.88, 95% CI = 1.19, 2.82) and nearly 4 times as many silver-haired bats (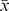 = 3.78, 95% CI = 1.12, 12.82; Tables 5 and 6) killed per control turbine than treatment turbine during the treatment period, these represented only about a 20% increase in fatality relative to the pre-treatment period. High variation among turbines, small numbers of carcasses found and frequent zero-counts of these and other species at each turbine prevented formal statistical tests of these ratios using the BACI design (Tables 6 and 7).

**Table 6:** Estimated bat fatalities/turbine (mean and 95% confidence intervals [CI]) for each species of bat per turbine, adjusted for searcher efficiency, carcass removal, and area, at control and treatment turbines at the Locust Ridge Wind Project in Columbia and Schuylkill Counties, Pennsylvania, 31 July–9 October 2010.

		Control Turbines				Treatment Turbines		
*Species*	N	Mean	Lower 95% CI	Upper 95% CI	N	Mean	Lower 95% CI	Upper 95% CI
Big brown bat	9	1.19	0.39	2.12	2	0.38	0.23	0.85
Eastern red bat	50	7.16	5.32	9.27	22	4.77	2.70	6.92
Hoary bat	61	9.12	7.08	11.70	22	5.02	3.37	7.31
Little brown bat	6	0.87	0.39	1.38	3	0.65	0.20	1.27
Silver-haired bat	20	2.87	1.48	4.47	5	1.00	0.18	2.03
Tri-colored bat	16	2.32	1.37	3.38	8	1.55	0.91	2.23

**Table 7:** Ratio between bat fatalities per control turbine relative to treatment turbines (mean and 95% confidence intervals [CI]) for each species of bat from the Locust Ridge Wind Project in Columbia and Schuylkill Counties, Pennsylvania, 31 July–9 October 2010.

Species	Mean Ratio control:deterrent	Lower 95% CI	Upper 95% CI
Big brown bat	3.72	0.70	7.87
Eastern red bat	1.59	0.93	2.78
Hoary bat*	1.88	1.19	2.82
Little brown bat	1.72	0.43	5.22
Silver-haired bat*	3.78	1.12	12.82
Tri-colored bat	1.59	0.84	2.96

Confidence intervals that do not include 1.0 are considered statistically significant (*).

## Discussion

Previous research has indicated difficulty when attempting to mask or “jam” bats’ echolocation except under specific conditions [e.g., 17, 33]. Indeed, bats can actually adjust their echolocation under jamming conditions [34], [35]. Bats are, however, likely “uncomfortable” when broadband ultrasound is present because it forces them to shift their call frequencies to avoid overlap, which in turn will lead to suboptimal use of echolocation or they may not echolocate at all [14], [34].

In contrast to previously tested acoustic “repellers” [36], the device we have developed and tested shows some promise for deterring bats from the surrounding airspace near wind turbines. This study represents the first field test of a deterrent device to reduce bat fatalities at wind turbines by comparing fatalities at treated and untreated turbines. Our findings generally corroborate our previous conclusions from unpublished laboratory and field experiments that a regime of presumably uncomfortable or disorienting ultrasound may deter bats from occupying such a treated airspace. While the treatment response we observed generally falls within the range of variation of fatalities among turbines we studied, nothing in the statistical evaluation of our data suggested that our random selection of the 10 treatment turbines somehow skewed mortality rates among the turbines we chose. We acknowledge that 3 of our treatment turbines had to be located on the Locust Ridge I portion of the facility where no control turbines were selected. While this could have influenced the results, we noted in 2009 that two of these three turbines had fewer mean fatalities relative to the overall mean for deterrent turbines, while in 2010, the mean fatalities of all three of these turbines generally were equal to or greater than the overall mean for treatment turbines. Fatalities at other turbines in both the control and treatment sets also varied from one year to the next and we do not believe data from the three turbines from LR I biased our findings.

In 2010, we examined potential inherent difference between the two sets of turbines and our findings suggested a marginal difference existed in fatalities between control and treatment turbines prior to implementation of the treatment. However, we caution that data from our pre-treatment period in 2010 was collected prior to migration of migratory tree roosting species and the ratio of migrant to non-migrant species was different between these two periods in our study. Thus, different levels of fatality, different species composition, and possibly different behaviors of the bats during the two phases may have influenced our findings regarding inherent differences between control and treatment turbines. Future field tests of deterrent devices should better account for potential differences in fatalities among different species when determining inherent variation among sample turbines.

The effectiveness of ultrasonic deterrents as a means to prevent bat fatalities at wind turbines is limited by the distance and area that ultrasound can be broadcast. Unfortunately, rapid attenuation of ultrasound in air, which is heavily influenced by humidity (Table S1), limits the effective range of broadcasts. Nightly humidity in this region of Pennsylvania averaged 86.5% in August 2009, 84.8% in September 2009, 80% in August 2010, and 76.8% in September 2010 (source http://climate.met.psu.edu/www_prod/). Assuming a constant temperature of 20°C and air pressure of 101.325 kPa and 80% humidity, the theoretical distance to “jam” bats at the assumed 65 dB level only extends to 20 m for the 20–30 kHz range, and declines to only 5–10 m for the upper frequency ranges of broadcast (70–100 kHz;). Ultrasound emission in the perpendicular plane of the rotor-swept area may be adequate to affect approaching bats, particularly those species influenced at the lower frequencies. However, it is clear that effective emissions in the parallel plane of the rotor-swept area will be difficult if not impossible to achieve based on sound attenuation in humid environments. The effective airspace would be different and larger in more arid environments, however (Table S1). We also note that some devices were not operating all the time during our study, due to malfunctions. Although we were unable to account for this factor in our analysis, clearly the affected airspace was reduced when some devices were inactive, which further influenced our findings.

We assume that when bats encounter a gradient of increasingly strong emissions as they approach the deterrent device, they will respond by flying opposite to that gradient to escape the effect of emissions. However, at present we know little about the general responses that various species of bats have upon entering a field of ultrasound emissions. It is therefore important to consider our assumptions when interpreting results of this study. Although our acoustic deterrent device could only generate a limited effective volume of uncomfortable airspace, bats could have detected the presence of such airspace from a greater range, possibly beyond the rotor swept area. Bats previously experiencing the discomfort of ultrasound broadcast may avoid approaching other treated towers, which they could detect as treated from beyond the zone of discomfort. In this way, ultrasound broadcast may effectively serve as acoustic beacons to direct bats away from wind turbines. Over time, bats may learn to avoid all turbines from their experience with those equipped with deterrents, similar to documented behavior of bats encountering other discomforting experiences such as mist nets. Just as bat capture success in mist nets declines on successive nights as bats apparently learn to detect the presence of nets and thereafter avoid them [37], [38], [39], [40], we speculate that after experiencing a disagreeable encounter with ultrasound treated airspace bats may opt to subsequently avoid it. Other lines of evidence indicate that bats learn and remember spatial locations or stimuli associated with obstacles or threats. A study that modified experiments conducted by Griffin [14] challenged bats to maneuver through vertical wires, and they did so by tilting and scrunching their wings; the same bats continued these maneuvers at the locations of wires even after they were removed [41]. In practice, the actual decline of activity at any treated site will likely depend upon immigration of naïve bats into the area. We did not monitor bat activity with night vision or thermal imaging cameras [42] and, thus, were unable to assess activity patterns of bats simultaneous with fatality searches. It is also possible that insects preyed on by bats in this region were deterred from the turbines, which could represent the ultimate cause of avoiding treated turbines. Indeed, studies have demonstrated that ultrasound can repel insects [43] and influence their reproduction [44]. However, we did not assess insect abundance and suggest future studies should attempt to address causal factors of avoidance including effect on insect prey.

Conversely, bats may habituate to the presence of ultrasound emissions and acoustic deterrents may actually lose their effectiveness over time. However, in prior field tests of deterrents, bats did not appear to habituate or accommodate to the presence of ultrasound emitted from a previous prototype deterrent at least over short periods of 5–7 days (J. Szewczak, Humboldt State University and E. Arnett, Bat Conservation International, unpublished data). Habituation to deterrents should, however, continue to be investigated in future studies.

The effectiveness of acoustic deterrents will likely vary among different species of bats. Hoary bats, for example, employ the lowest frequency range of the species we studied (∼20–25 kHz) and may be affected more so than other species that use higher frequencies and perhaps fly at further distances from the device. Hoary bats had significantly fewer fatalities at turbines with deterrents relative to those without them in both years, and silver-haired bats also had fewer fatalities at turbines with deterrents in 2010. In 2010, however, after accounting for inherent differences between turbine sets prior to treatment, hoary and silver-haired bats killed per control turbine relative to treatment turbines during the treatment period represented only a 20% increase in fatality over the pre-treatment period. Species-specific effectiveness warrants further investigation in a study with more power to detect differences among species. Future studies hopefully will also elucidate whether deterrents can eventually serve as a mitigation tool for minimizing or eliminating take of threatened or endangered species such as the Indiana bat (*Myotis sodalis*). The limited range of ultrasound broadcast from a wind turbine tower or nacelle might have only a moderate contribution toward reducing impacts of bats randomly flying through the rotor-swept area. However, for bats that may be drawn to and approach turbine towers as potential roosts or gathering sites [1], [45], the combination of effective range and learned avoidance response to ultrasound broadcast may have longer term effects in reducing bat mortality at wind turbines. We also note that we only tested broadband ultrasound emission (20–100 kHz) and short pulses mimicking echoes of insects [16], for example, could prove to be more effective for some species and should be tested in future studies.

Introducing ultrasound emissions into the environment could potentially yield negative environmental effects on other species of wildlife, but we do not feel this is of concern because the device we tested only had a limited effective range because of rapid attenuation of ultrasound with distance from its source. Within the effective range of the treated airspace, emissions could affect ultrasound-sensitive ) insects and disperse them, providing less reason for bats to occupy that airspace, assuming food sources attract bats to turbines [1], [10]; and 2) passerines that may be deterred from the airspace, thus reducing strikes. If ultrasound could reach the ground from locations where deterrent devices are mounted, which was not the case in our study, ultrasound sensitive small mammals could be dispersed away from the base of the turbines, but this would likely be a positive impact that could reduce strikes of stooping raptors.

### Conclusions

This study, and previous experiments with earlier prototypes, revealed that broadband ultrasound broadcasts may affect bat behavior directly by discouraging them from approaching the sound source, or indirectly by reducing the time bats spend foraging near a turbine if insects are repelled by ultrasound [e.g., 42, 43; also recognizing not all insects have ears to detect ultrasound] and ultimately reduce bat fatalities at wind turbines. However, variation among turbines yielded inconclusive evidence of a strong effect of deterrents on bat fatality and while the approach may hold some promise, further refinement and investigation is needed. We did experience technical issues in both years of the study, including water leakage that rendered some deterrents inoperable during portions of the study period which clearly influenced our findings. Thus, results from this study may reflect a more conservative estimate of potential fatality reduction achievable through application of the deterrent device we tested. Still, we caution that the response estimated in this study falls generally within the range of variation for bat fatalities among turbines in this and other studies in the region [2]. Additionally, deterrents resulted in lower reductions in bat fatality relative to curtailing turbine operations by increasing cut-in speeds (44–93%) [11], [12]. We further caution that it would be premature and unwarranted to conclude or interpret from these initial results that this technology provides an operational deterrent device ready for broad-scale deployment at wind facilities. While we do not consider acoustic deterrents to be an acceptable mitigation strategy at this time, with further experimentation and modifications, this type of deterrent method may prove successful and broadly applicable for protecting bats from harmful encounters with wind turbine blades. Future research and development and field studies should attempt to improve the device and it’s weatherproofing and emission performance, and optimizes the placement and number of devices on each turbine that would affect the greatest amount of airspace in the rotor-swept area to estimate potential maximum effectiveness of this tool to reduce bat fatalities. New studies also should test other emission types such as short ultrasonic pulses that mimic insects [16]. Finally, we did not attempt to develop comparative estimates of costs associated with our deterrent devices relative to lost revue of operational mitigation because current deterrent development costs are high and dynamic and operational costs to maintain them over a period of time have not been established. Future efforts should determine production and maintenance costs of newly developed deterrents that can be factored into a cost-benefit analysis comparing different approaches for mitigating bat fatalities.

## Supporting Information

Figure S1 **An ultrasonic deterrent device used in this study (Photo by E. Arnett, Bat Conservation International).**
(JPG)

Figure S2 **Ultrasonic deterrent devices mounted on the side of the turbine nacelle (photo by E. Arnett, Bat Conservation International).**
(JPG)

Figure S3 **Depiction of acoustic deterrent placement on the nacelle of turbines and ultrasonic broadcast volume from devices (broadcast volume approximation of data from Senscorp beam pattern data, see supplemental material below).**
(DOCX)

Figure S4 **Hypothetical carcass search plot for a wind turbine illustrating 2 m rings extending from the turbine edge out to the theoretical maximum plot distance and a depiction of “easy” searchable area (shaded area within line drawing) in the plot, used to develop weights for adjusting fatalities.**
(DOCX)

Table S1 **Calculated decibel level at different distances and frequencies at two different levels of relative humidity (10 and 40%) for acoustic deterrent devices used in this study.** Calculations assume ambient temperature of 20°C and air pressure of 101.325 kPa (kilopascal).
(DOCX)

Table S2 **The attenuation of sound in air due to viscous, thermal and rotational loss mechanisms is simply proportional to** ***f*** **^2^.** However, losses due to vibrational relaxation of oxygen molecules are generally much greater than those due to the classical processes, and the attenuation of sound varies significantly with temperature, water-vapor content and frequency. A method for calculating the absorption at a given temperature, humidity, and pressure can be found in ISO 9613-1 (1993). The table and figure below gives values of attenuation in dB m^−1^ for a temperature of 20°C and an air pressure of 101.325 kPa. The uncertainty is estimated to be ±10%.
(DOCX)

Table S3 **Habitat visibility classes used during this study, following Pennsylvania Game Commission Protocol** [**21**] **.** Data for Classes 3 and 4 were combined during our final analyses.
(DOCX)

## References

[1] Kunz TH, Arnett EB, Erickson WP, Johnson GD, Larkin RP, et al (2007) Ecological impacts of wind energy development on bats: questions, hypotheses, and research needs. Front Ecol Environ 5: 315–324.

[2] Arnett EB, Brown K, Erickson WP, Fiedler J, Henry TH, et al (2008) Patterns of fatality of bats at wind energy facilities in North America. J Wildl Manage 72: 61–78.

[3] Baerwald EF, Barclay RMR (2009) Geographic variation in activity and fatality of migratory bats at wind energy facilities. J Mammal 90: 1341–1349.

[4] Rydell J, Bach L, Dubourg-Savage M, Green M, Rodrigues L, et al (2010) Bat mortality at wind turbines in northwestern Europe. Acta Chiropt 12: 261–274.

[5] Arnett EB, Baerwald EF (2013) Impacts of wind energy development on bats: implications for conservation. In: Adams RA, Peterson SC, editors. Bat evolution, ecology, and conservation. New York: Springer Science Press (in press).

[6] Energy Information Administration. (2010) Annual energy outlook 2010 with projections to 2035. U.S. Department of Energy, Energy Information Administration website. Available: http://www.eia.doe.gov/oiaf/ieo/world.html. Accessed 2011 December 15.

[7] Racey PA, Entwistle AC (2003) Conservation ecology of bats. In: Kunz TH, Fenton MB, editors. Bat Ecology. Chicago: University of Chicago Press. 680–743.

[8] Winhold L, Kurta A, Foster R (2008) Long-term change in an assemblage of North American bats: are eastern red bats declining? Acta Chiropta 10: 359–366).

[9] Frick WF, Pollock JF, Hicks AC, Langwig KE, Reynolds DS, et al (2010) An emerging disease causes regional population collapse of a common North American bat species. Science 329: 679–682. 20689016 10.1126/science.1188594

[10] Cryan PM, Barclay RMR (2009) Causes of bat fatalities at wind turbines: hypotheses and predictions. J Mammal 90: 1330–1340.

[11] Baerwald EF, Edworthy J, Holder M, Barclay RMR (2009) A large-scale mitigation experiment to reduce bat fatalities at wind energy facilities. J Wildl Manage 73: 1077–1081.

[12] Arnett EB, Huso MMP, Schirmacher MR, Hayes JP (2011) Changing wind turbine cut-in speed reduces bat fatalities at wind facilities. Front Ecol Environ 9: 209–214.

[13] Nicholls B, Racey PA (2009) The aversive effect of electromagnetic radiation on foraging bats–a possible means of discouraging bats from approaching wind turbines. PLoS ONE 4(7): e6246 doi:10.1371/journal.pone.0006246 19606214 10.1371/journal.pone.0006246 PMC2705803

[14] Griffin DR (1958) Listening in the dark. New Haven: Yale University Press. 413 p.

[15] Barlow KE, Jones G (1997) Function of pipistrelle social calls: field data and a playback experiment. Animal Behaviour 53: 991–999.

[16] Hristov NI, Conner WE (2005) Sound strategy: acoustic aposematism in the bat-tiger moth arms race. Naturwissenschaften 92: 164–169. 15772807 10.1007/s00114-005-0611-7

[17] Griffin DR, McCue JJG, Grinnell AD (1963) The resistance of bats to jamming. J Exp Zool 152: 229–250.

[18] Mackey RL, Barclay RMR (1989) The influence of physical clutter and noise on the activity of bats over water. Can J Zool 67: 1167–1170.

[19] Brown RG, Brown ML (1972) Woody Plants of Maryland. Baltimore: Port City Press 347 p.

[20] Strausbaugh PD, Core EL (1978) Flora of West Virginia. Second edition. Grantsville: Seneca Books, 1079 p.

[21] Pennsylvania Game Commission (2007) Pennsylvania Game Commission wind energy voluntary cooperation agreement. Pennsylvania Game Commission website. Available: http://www.portal.state.pa.us/portal/server.pt/gateway/PTARGS_0_0_114831_0_0_43/http/pubcontent.state.pa.us/publishedcontent/publish/marketingsites/game_commission/content/wildlife/habitat_management/wind_energy/wind_energy_voluntary_cooperative_agreement_ci.html?qid=85232671&rank=6. Accessed 2013 April 24.

[22] Surlykke A, Kalko EKV (2008) Echolocating bats cry out loud to detect their prey. PLoS ONE 3(4): e2036 doi:10.1371/journal.pone.0002036 18446226 10.1371/journal.pone.0002036 PMC2323577

[23] Fenton MB (2003) Eavesdropping on the echolocation and social calls of bats. Mammal Rev 33: 193–204.

[24] Parson S, Szewczak JM (2009) Detecting, recording, and analyzing the vocalizations of bats. In: Kunz TH, Parsons S, editors. Ecological and behavioral methods for the study of bats, 2^nd^ edition. Baltimore: Johns Hopkins University Press. 901 p.

[25] Gannon WL, Sikes RS (2007) the Animal Care and Use Committee of the American Society of Mammalogists (2007) Guidelines of the American Society of Mammalogists for the use of wild mammals in research. J Mammal 88: 809–823. 10.1093/jmammal/gyw078 PMC5909806 29692469

[26] Strickland MD, Arnett EB, Erickson WP, Johnson DH, Johnson GD, et al. (2011) Comprehensive guide to studying wind energy/wildlife interactions. National Wind Coordinating Collaborative website. Available: http://www.nationalwind.org/assets/publications/Comprehensive_Guide_to_Studying_Wind_Energy_Wildlife_Interactions_2011_Updated.pdf. Accessed 2013 April 24.

[27] Huso MMP (2011) An estimator of wildlife fatality from observed carcasses. Environmetrics 22: 318–329.

[28] Cochran WG (1977) Sampling techniques, 3rd edition. New York: John Wiley & Sons. 428 p.

[29] Manly BFJ (1997) Randomization and Monte Carlo Methods in Biology, 2^nd^ edition. New York: Chapman and Hall. 300 p.

[30] Hurlbert SH (1984) Pseudoreplication and the design of ecological field experiments. Ecol Monogr 54: 187–211.

[31] Hewitt JE, Thrush SE, Cummings VJ (2001) Assessing environmental impacts: effects of spatial and temporal variability at likely impact scales. Ecol Appl 11: 1502–1516.

[32] Steele RGD, Torrie JH, Dickie DA (1997) Principles and procedures of statistics: a biometrical approach, 3^rd^ edition. Boston: McGraw-Hill. 666 p.

[33] Møhl B, Surlykke A (1989) Detection of sonar signals in the presence of pulses of masking noise by the echolocating bat, *Eptesicus fuscus* . J Comp Physiol 165: 119–194.

[34] Ulanovsky N, Fenton MB, Tsoar A, Korine C (2004) Dynamics of jamming avoidance in echolocating bats. Proceed Royal Soc London B 271: 1467–1475. 10.1098/rspb.2004.2750 PMC1691745 15306318

[35] Gillam EH, McCracken GF (2007) Variability in the echolocation of *Tadarida brasiliensis*: effects of geography and local acoustic environment. Animal Behav 74: 277–286.

[36] Hurley S, Fenton MB (1980) Ineffectiveness of fenthion, zinc phosphide, DDT and two ultrasonic rodent repellers for control of populations of little brown bats (*Myotis lucifugus*). Bull Environ Contamin and Toxicol 25: 503–507. 10.1007/BF01985562 7426802

[37] Kunz TH, Hodgkison R, Weise C (2009) Methods for capturing and handling bats. In: Kunz TH, Parsons s, editors. Ecological and behavioral methods for the study of bats, 2^nd^ edition. Baltimore: Johns Hopkins University Press. 1–35.

[38] Larsen RJ, Boegler KA, Genoways HH, Masefield WP, Kirsch RA, et al (2007) Mist netting bias, species accumulation curves, and the rediscovery of two bats on Montserrat (Lesser Antilles). Acta Chiropt 9: 423–435.

[39] Robbins LW, Murray KL, McKenzie PM (2008) Evaluating the effectiveness of the standard mist-netting protocol for the endangered Indiana bat (*Myotis sodalis*). Northeast. Nat. 15: 275–282.

[40] Winhold L, Kurta A (2008) Netting surveys for bats in the northeast: differences associated with habitat, duration of netting, and use of consecutive nights. Northeast. Nat. 15: 263–274.

[41] Pollak GD, Casseday JH (1989). The Neural Basis of Echolocation in Bats. Berlin: Springer-Verlag. 155 p.

[42] Horn J, Arnett EB, Kunz TH (2008) Behavioral responses of bats to operating wind turbines. J Wildl Manage 72: 123–132.

[43] Belton P, Kempster RH (1962) A field test on the use of sound to repel the European corn borer. Entomologia 5: 281–288.

[44] Huang F, Subramanyam B, Taylor R (2011) Ultrasound affects spermatophore transfer, larval numbers, and larval weight of *Plodia interpunctella* (Hübner) (Lepidoptera: Pyralidae). J Stored Products Res 39: 413–422.

[45] Cryan PM (2008) Mating behavior as a possible cause of bat fatalities at wind turbines. J Wildl Manage 72: 845–849.

