# Correction: Evaluating the Effectiveness of an Ultrasonic Acoustic Deterrent for Reducing Bat Fatalities at Wind Turbines

**DOI:** 10.1371/annotation/a81f59cb-0f82-4c84-a743-895acb4b2794

**Published:** 2013-09-10

**Authors:** Edward B. Arnett, Cris D. Hein, Michael R. Schirmacher, Manuela M. P. Huso, Joseph M. Szewczak

The following phrase was erroneously omitted from the Competing Interests statement: Any use of trade, product, or firm names is for descriptive purposes only and does not imply endorsement by the U.S. Government.

There was also an error in Manuela Huso's affiliation. The correct affiliation is: Manuela M. P. Huso, Forest and Rangeland Ecosystem Science Center, U.S. Geological Survey, Corvallis, Oregon, United States of America. 

