# Supplementary figures and images for: Evaluating the Effectiveness of an Ultrasonic Acoustic Deterrent for Reducing Bat Fatalities at Wind Turbines
Source: PLoS One. 2013 Jun 19;8(6):e65794. doi: 10.1371/journal.pone.0065794 (PMC3686786; doi:10.1371/journal.pone.0065794)

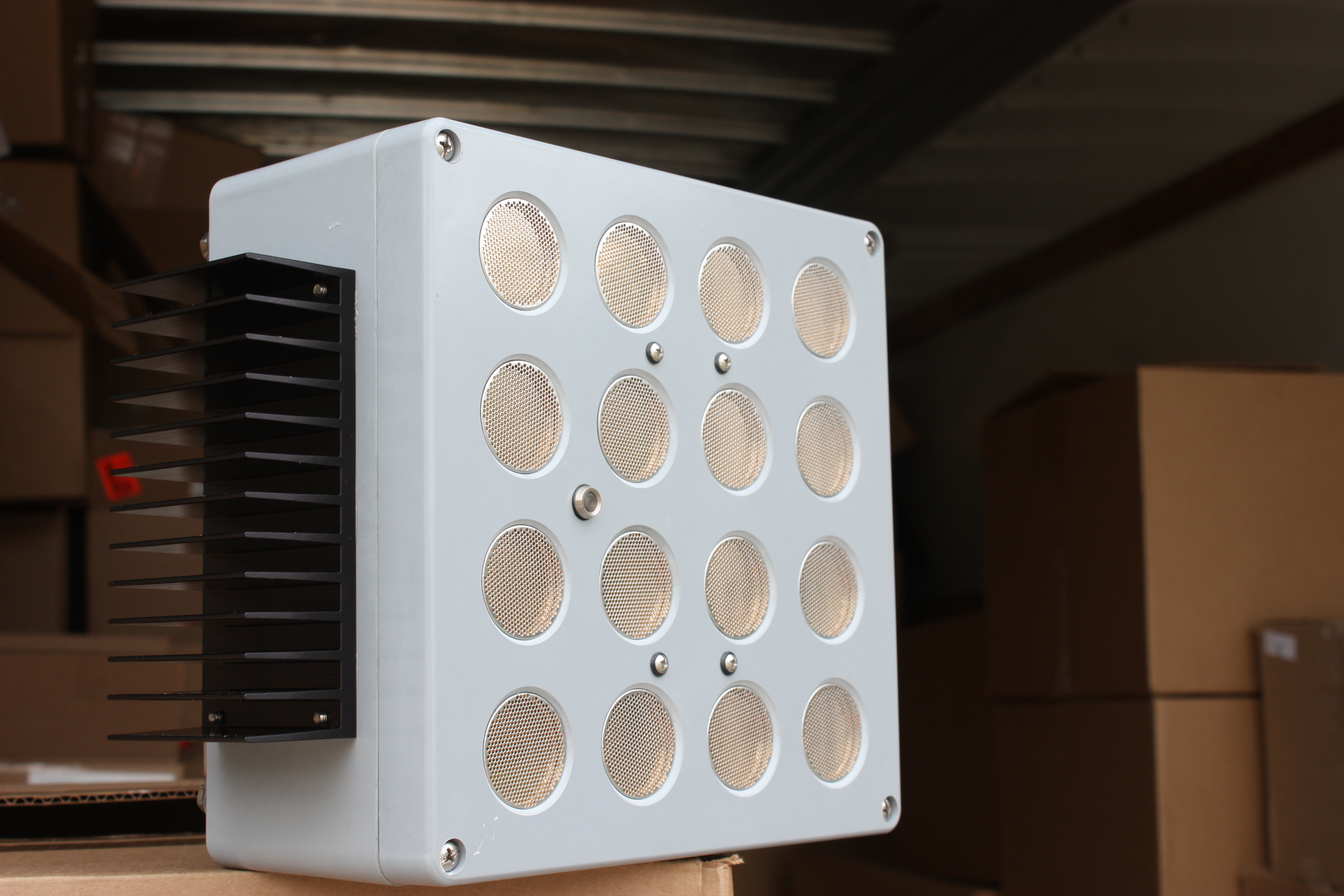

Supplement: Figure S1 — An ultrasonic deterrent device used in this study (Photo by E. Arnett, Bat Conservation International). (JPG) [file pone.0065794.s001.jpg]

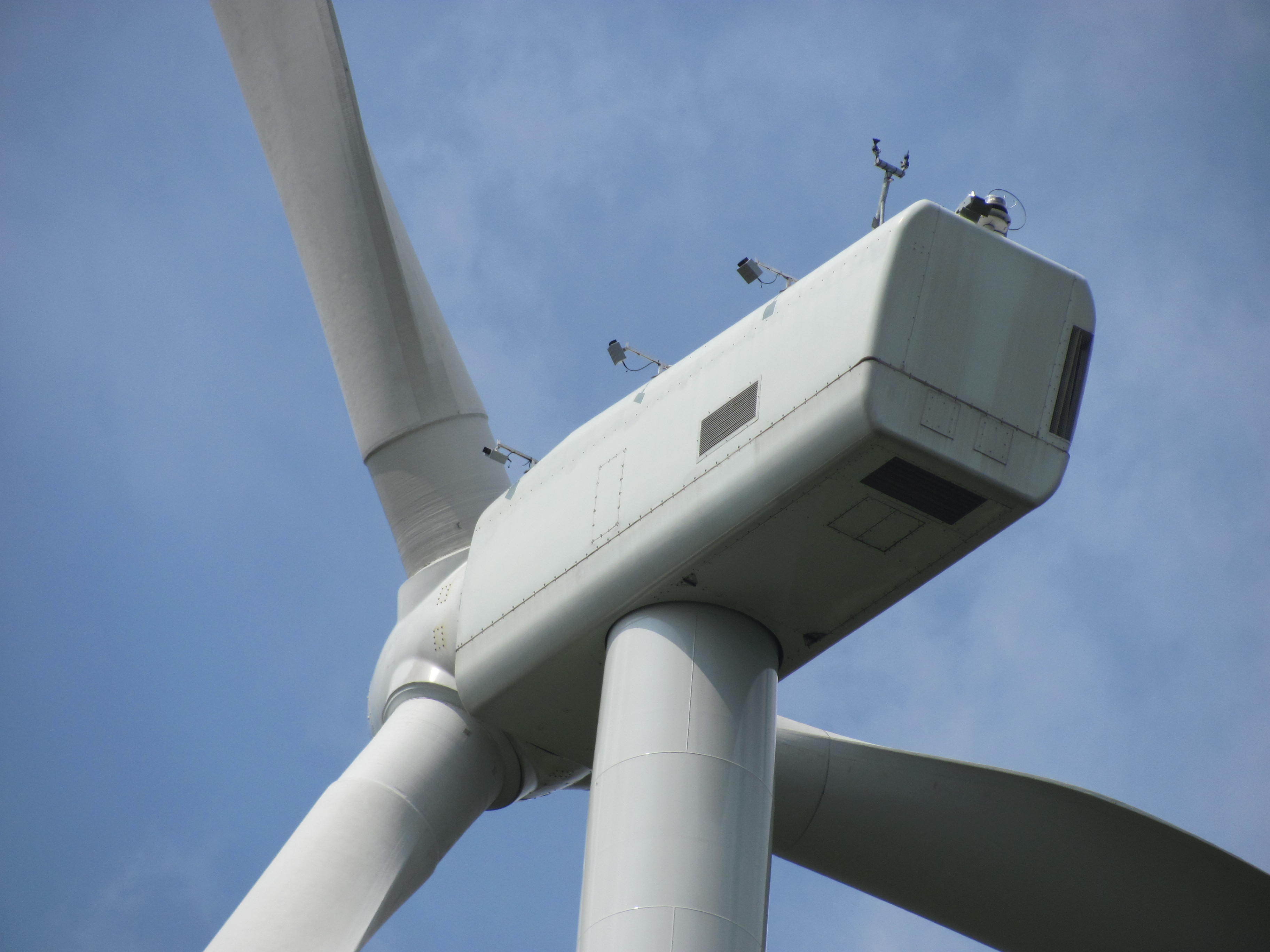

Supplement: Figure S2 — Ultrasonic deterrent devices mounted on the side of the turbine nacelle (photo by E. Arnett, Bat Conservation International). (JPG) [file pone.0065794.s002.jpg]
