# Supplementary material for: Evaluating the Effectiveness of an Ultrasonic Acoustic Deterrent for Reducing Bat Fatalities at Wind Turbines
Source: PLoS One. 2013 Jun 19;8(6):e65794. doi: 10.1371/journal.pone.0065794 (PMC3686786; doi:10.1371/journal.pone.0065794)
Supplement: Figure S3 — Depiction of acoustic deterrent placement on the nacelle of turbines and ultrasonic broadcast volume from devices (broadcast volume approximation of data from Senscorp beam pattern data, see supplemental material below). (DOCX) [file pone.0065794.s003.docx]

**Figure S3.** Depiction of acoustic deterrent placement on the nacelle of turbines and ultrasonic broadcast volume from devices (broadcast volume approximation of data from Senscorp beam pattern data, see supplemental material below).

**
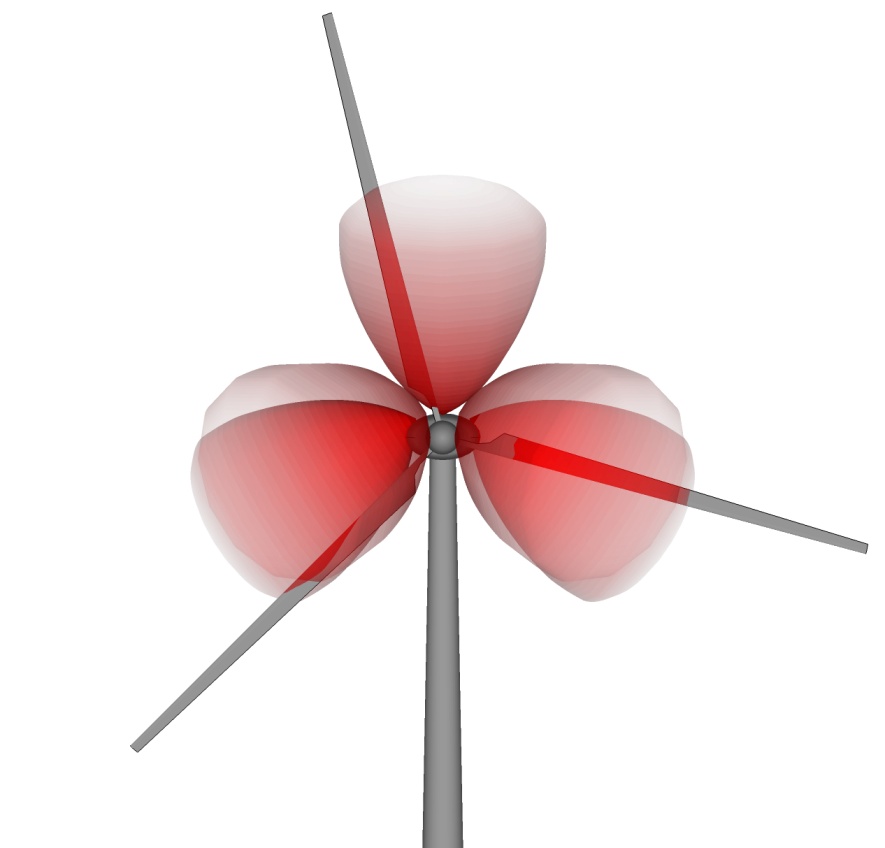
**

**
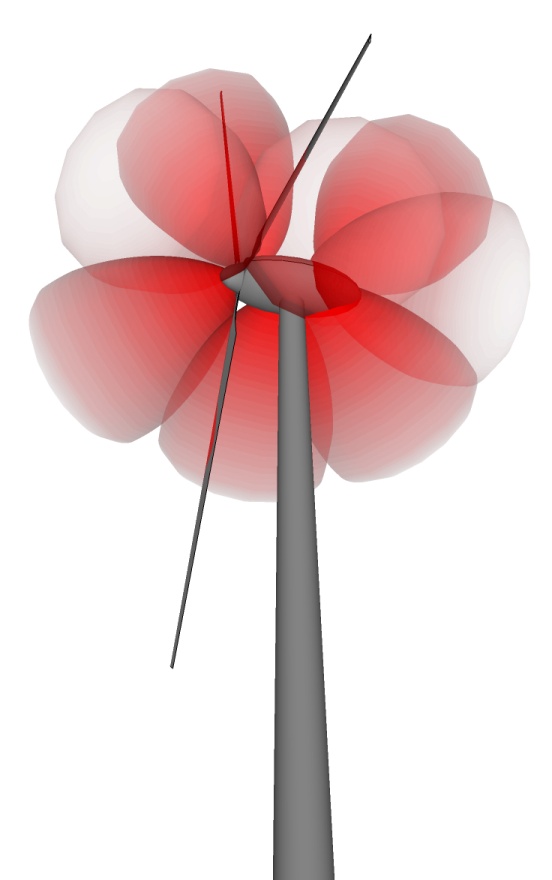
**
