# Supplementary material for: Evaluating the Effectiveness of an Ultrasonic Acoustic Deterrent for Reducing Bat Fatalities at Wind Turbines
Source: PLoS One. 2013 Jun 19;8(6):e65794. doi: 10.1371/journal.pone.0065794 (PMC3686786; doi:10.1371/journal.pone.0065794)

**Figure S-4.** Hypothetical carcass search plot for a wind turbine illustrating 2 m rings extending from the turbine edge out to the theoretical maximum plot distance and a depiction of “easy” searchable area (shaded area within line drawing) in the plot, used to develop weights for adjusting fatalities.


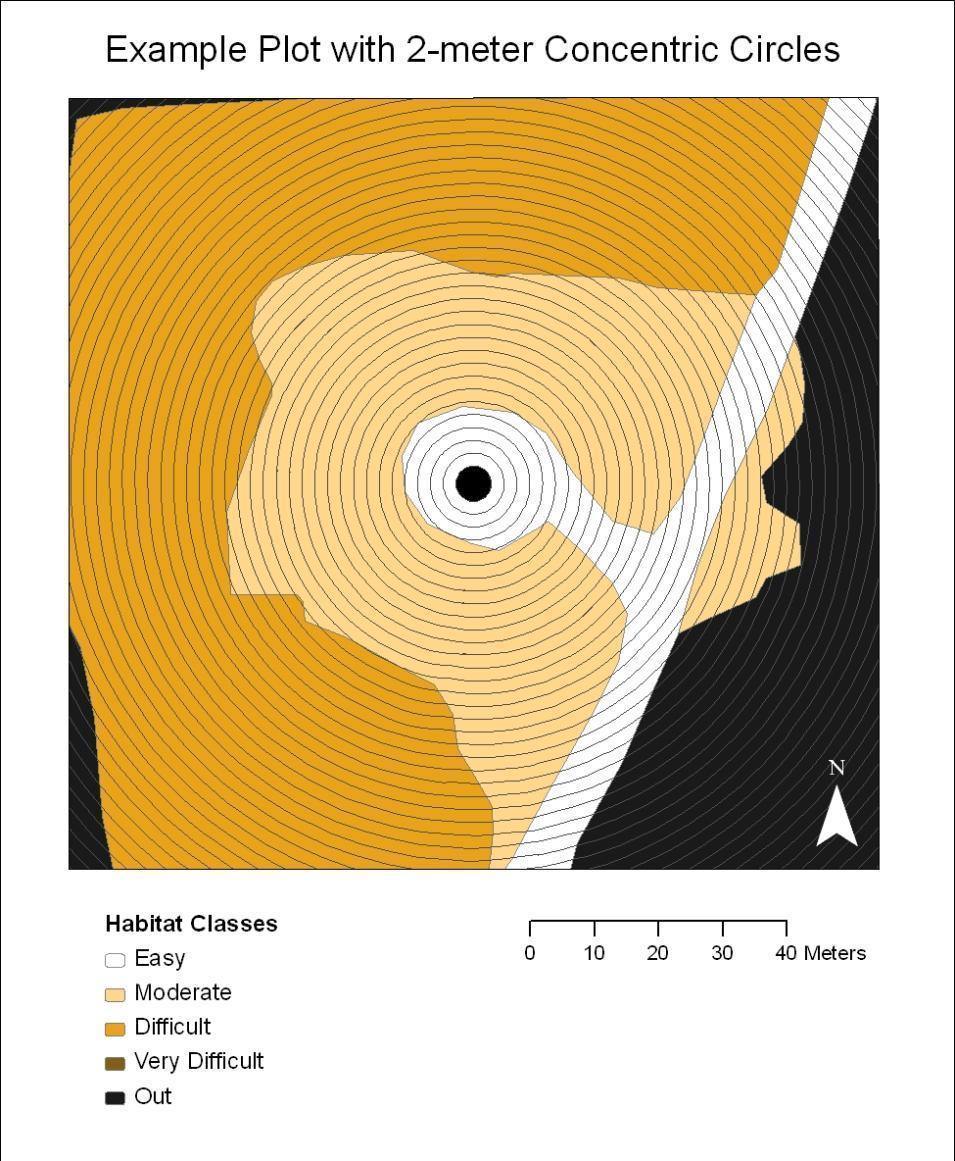

Supplement: Figure S4 — Hypothetical carcass search plot for a wind turbine illustrating 2 m rings extending from the turbine edge out to the theoretical maximum plot distance and a depiction of “easy” searchable area (shaded area within line drawing) in the plot, used to develop weights for adjusting fatalities. (DOCX) [file pone.0065794.s004.docx]
