# Supplementary material for: Evaluating the Effectiveness of an Ultrasonic Acoustic Deterrent for Reducing Bat Fatalities at Wind Turbines
Source: PLoS One. 2013 Jun 19;8(6):e65794. doi: 10.1371/journal.pone.0065794 (PMC3686786; doi:10.1371/journal.pone.0065794)
Supplement: Table S1 — Calculated decibel level at different distances and frequencies at two different levels of relative humidity (10 and 40%) for acoustic deterrent devices used in this study. Calculations assume ambient temperature of 20°C and air pressure of 101.325 kPa (kilopascal). (DOCX) [file pone.0065794.s005.docx]

**Table S1.** Calculated decibel level at different distances and frequencies at two different levels of relative humidity (10 and 40%) for acoustic deterrent devices used in this study. Calculations assume ambient temperature of 20^o^ C and air pressure of 101.325 kPa (kilopascal).

| **Calculated Decibel Level at Distance and Frequency** | | | | | | | | | |
| --- | --- | --- | --- | --- | --- | --- | --- | --- | --- |
| **(Assumes 20^o^ C at 10% relative humidity and pressure of 101.325 kPa)** | | | | | | | | | |
|  | **Frequency (kHz)** | | | | | | | | |
| **Distance (m)** | **20** | **30** | **40** | **50** | **60** | **70** | **80** | **90** | **100** |
| **1** | 102 | 107 | 112 | 122 | 122 | 117 | 114.5 | 114.5 | 117 |
| **5** | 87.0 | 91.6 | 96.2 | 105.6 | 104.7 | 99.1 | 95.7 | 94.5 | 95.8 |
| **10** | 79.7 | 83.9 | 87.9 | 96.6 | 94.4 | 88.1 | 83.7 | 81.0 | 80.8 |
| **15** | 74.8 | 78.7 | 82.0 | 90.1 | 86.7 | 79.7 | 74.2 | 70.0 | 68.3 |
| **20** | 71.0 | 74.5 | 77.2 | 84.6 | 80.0 | 72.3 | 65.7 | 60.0 | 56.8 |
| **25** | 67.8 | 70.8 | 73.0 | 79.6 | 73.9 | 65.4 | 57.7 | 50.6 | 45.8 |
| **30** | 64.9 | 67.5 | 69.1 | 75.0 | 68.1 | 58.9 | 50.2 | 41.6 | 35.3 |
| **35** | 62.3 | 64.5 | 65.5 | 70.7 | 62.6 | 52.6 | 42.8 | 32.7 | 24.9 |
| **40** | 59.8 | 61.6 | 62.0 | 66.5 | 57.2 | 46.5 | 35.7 | 24.1 | 14.8 |
| **45** | 57.5 | 58.8 | 58.7 | 62.5 | 52.0 | 40.6 | 28.6 | 15.6 | 4.7 |
| **50** | 55.3 | 56.2 | 55.5 | 58.6 | 46.9 | 34.8 | 21.7 | 7.2 | -5.2 |
| **55** | 53.2 | 53.7 | 52.4 | 54.7 | 41.8 | 29.0 | 14.9 | -1.1 | -15.0 |
| **60** | 51.1 | 51.2 | 49.3 | 51.0 | 36.9 | 23.3 | 8.1 | -9.4 | -24.8 |
|  |  |  |  |  |  |  |  |  |  |
| **Calculated Decibel Level at Distance and Frequency** | | | | | | | | | |
| **(Assumes 20^o^ C at 40% relative humidity and pressure of 101.325 kPa)** | | | | | | | | | |
|  | **Frequency (kHz)** | | | | | | | | |
| **Distance (m)** | **20** | **30** | **40** | **50** | **60** | **70** | **80** | **90** | **100** |
| **1** | 102 | 107 | 112 | 122 | 122 | 117 | 114.5 | 114.5 | 117 |
| **5** | 85.7 | 89.3 | 93.2 | 102.0 | 100.8 | 94.9 | 91.3 | 90.1 | 91.4 |
| **10** | 76.8 | 78.5 | 81.2 | 88.4 | 85.8 | 78.7 | 73.8 | 71.0 | 70.9 |
| **15** | 70.4 | 70.3 | 71.7 | 77.3 | 73.3 | 65.0 | 58.8 | 54.5 | 52.9 |
| **20** | 65.0 | 63.1 | 63.2 | 67.2 | 61.8 | 52.4 | 44.8 | 38.9 | 35.9 |
| **25** | 60.1 | 56.4 | 55.2 | 57.8 | 50.8 | 40.3 | 31.3 | 23.9 | 19.4 |
| **30** | 55.6 | 50.2 | 47.7 | 48.6 | 40.3 | 28.5 | 18.3 | 9.3 | 3.4 |
| **35** | 51.4 | 44.1 | 40.3 | 39.7 | 29.9 | 17.0 | 5.4 | -5.1 | -12.5 |
| **40** | 47.3 | 38.2 | 33.2 | 31.0 | 19.8 | 5.7 | -7.2 | -19.3 | -28.1 |
| **45** | 43.4 | 32.5 | 26.1 | 22.4 | 9.7 | -5.5 | -19.8 | -33.4 | -43.7 |
| **50** | 39.6 | 26.9 | 19.2 | 13.9 | -0.2 | -16.5 | -32.2 | -47.3 | -59.1 |
| **55** | 35.9 | 21.3 | 12.4 | 5.5 | -10.0 | -27.5 | -44.5 | -61.2 | -74.4 |
| **60** | 32.2 | 15.9 | 5.6 | -2.8 | -19.8 | -38.4 | -56.8 | -75.0 | -89.7 |

| Upper Target (dB) | 65 |
| --- | --- |
| lower Trarget (dB) | 35 |

**Table SM-1.** - continued.

| **Calculated Decibel Level at Distance and Frequency** | | | | | | | | | |
| --- | --- | --- | --- | --- | --- | --- | --- | --- | --- |
| **(Assumes 20^o^ C at 80% relative humidity and pressure of 101.325 kPa)** | | | | | | | | | |
|  | **Frequency (kHz)** | | | | | | | | |
| **Distance (m)** | **20** | **30** | **40** | **50** | **60** | **70** | **80** | **90** | **100** |
| **1** | 102 | 107 | 112 | 122 | 122 | 117 | 114.5 | 114.5 | 117 |
| **5** | 86.5 | 89.9 | 93.2 | 101.2 | 98.8 | 92.4 | 88.1 | 86.3 | 87.0 |
| **10** | 78.6 | 80.0 | 81.2 | 86.6 | 81.3 | 73.2 | 66.6 | 62.6 | 61.0 |
| **15** | 73.2 | 72.6 | 71.7 | 74.6 | 66.3 | 56.5 | 47.6 | 41.3 | 37.5 |
| **20** | 68.8 | 66.2 | 63.2 | 63.5 | 52.3 | 40.8 | 29.6 | 21.1 | 15.0 |
| **25** | 64.9 | 60.4 | 55.2 | 53.1 | 38.8 | 25.6 | 12.1 | 1.4 | -7.0 |
| **30** | 61.4 | 55.0 | 47.7 | 42.9 | 25.8 | 10.8 | -4.9 | -17.9 | -28.5 |
| **35** | 58.2 | 49.8 | 40.3 | 33.1 | 12.9 | -3.7 | -21.8 | -36.9 | -49.9 |
| **40** | 55.1 | 44.7 | 33.2 | 23.4 | 0.3 | -18.1 | -38.4 | -55.8 | -71.0 |
| **45** | 52.2 | 39.8 | 26.1 | 13.8 | -12.3 | -32.3 | -55.0 | -74.6 | -92.1 |
| **50** | 49.4 | 35.0 | 19.2 | 4.4 | -24.7 | -46.5 | -71.4 | -93.2 | -113.0 |
| **55** | 46.7 | 30.3 | 12.4 | -5.0 | -37.0 | -60.5 | -87.7 | -111.8 | -133.8 |
| **60** | 44.0 | 25.7 | 5.6 | -14.3 | -49.3 | -74.5 | -104.0 | -130.2 | -154.6 |

| Upper Target (dB) | 65 |
| --- | --- |
| lower Trarget (dB) | 35 |
