# Supplementary material for: Evaluating the Effectiveness of an Ultrasonic Acoustic Deterrent for Reducing Bat Fatalities at Wind Turbines
Source: PLoS One. 2013 Jun 19;8(6):e65794. doi: 10.1371/journal.pone.0065794 (PMC3686786; doi:10.1371/journal.pone.0065794)
Supplement: Table S2 — The attenuation of sound in air due to viscous, thermal and rotational loss mechanisms is simply proportional to f 2. However, losses due to vibrational relaxation of oxygen molecules are generally much greater than those due to the classical processes, and the attenuation of sound varies significantly with temperature, water-vapor content and frequency. A method for calculating the absorption at a given temperature, humidity, and pressure can be found in ISO 9613-1 (1993). The table and figure below gives values of attenuation in dB m−1 for a temperature of 20°C and an air pressure of 101.325 kPa. The uncertainty is estimated to be ±10%. (DOCX) [file pone.0065794.s006.docx]

**Table SM-2.** The attenuation of sound in air due to viscous, thermal and rotational loss mechanisms is simply proportional to *f* ^2^. However, losses due to vibrational relaxation of oxygen molecules are generally much greater than those due to the classical processes, and the attenuation of sound varies significantly with temperature, water-vapor content and frequency. A method for calculating the absorption at a given temperature, humidity, and pressure can be found in ISO 9613-1 (1993). The table and figure below gives values of attenuation in dB m^−1^ for a temperature of 20° C and an air pressure of 101.325 kPa. The uncertainty is estimated to be ± 10%.

| **Absorption Coefficient (per ISO9613-1) at 20C and pressure of 101.325 kPa** | | | | | | | | | |
| --- | --- | --- | --- | --- | --- | --- | --- | --- | --- |
|  | **Relative Humidity** | | | | | | | | |
| **Frequency** | **10** | **20** | **30** | **40** | **50** | **60** | **70** | **80** | **90** |
| **20** | 0.26 | 0.51 | 0.60 | 0.58 | 0.52 | 0.47 | 0.42 | 0.38 | 0.35 |
| **30** | 0.34 | 0.65 | 0.86 | 0.94 | 0.94 | 0.89 | 0.83 | 0.78 | 0.72 |
| **40** | 0.46 | 0.78 | 1.10 | 1.20 | 1.30 | 1.30 | 1.30 | 1.20 | 1.20 |
| **50** | 0.60 | 0.94 | 1.27 | 1.51 | 1.66 | 1.73 | 1.74 | 1.71 | 1.66 |
| **60** | 0.84 | 1.20 | 1.50 | 1.80 | 2.10 | 2.20 | 2.30 | 2.30 | 2.30 |
| **70** | 0.98 | 1.33 | 1.70 | 2.03 | 2.29 | 2.47 | 2.59 | 2.64 | 2.66 |
| **80** | 1.20 | 1.60 | 2.00 | 2.30 | 2.60 | 2.80 | 3.00 | 3.10 | 3.10 |
| **90** | 1.50 | 1.85 | 2.24 | 2.61 | 2.93 | 3.20 | 3.40 | 3.55 | 3.64 |
| **100** | 1.80 | 2.20 | 2.50 | 2.90 | 3.30 | 3.60 | 3.80 | 4.00 | 4.10 |
