# Supplementary material for: Evaluating the Effectiveness of an Ultrasonic Acoustic Deterrent for Reducing Bat Fatalities at Wind Turbines
Source: PLoS One. 2013 Jun 19;8(6):e65794. doi: 10.1371/journal.pone.0065794 (PMC3686786; doi:10.1371/journal.pone.0065794)
Supplement: Table S3 — Habitat visibility classes used during this study, following Pennsylvania Game Commission Protocol [21] . Data for Classes 3 and 4 were combined during our final analyses. (DOCX) [file pone.0065794.s007.docx]

**Table S3.** Habitat visibility classes used during this study, following Pennsylvania Game Commission Protocol [21]. Data for Classes 3 and 4 were combined during our final analyses.

| **% Vegetative Cover** | **Vegetation Height** | **Visibility Class** |
| --- | --- | --- |
| >90% bare ground | <15 cm tall | Class 1 (Easy) |
|  |  |  |
| >25% bare ground | <15 cm tall | Class 2 (Moderate) |
|  |  |  |
| <25% bare ground | <25% > 30 cm tall | Class 3 (Difficult) |
|  |  |  |
| Little or no bare ground | >25% > 30 cm tall | Class 4 (Very Difficult) |
|  |  |  |
